# Experimental Study of Long Short-Term Memory and Transformer Models for Fall Detection on Smartwatches

**DOI:** 10.3390/s24196235

**Published:** 2024-09-26

**Authors:** Syed Tousiful Haque, Minakshi Debnath, Awatif Yasmin, Tarek Mahmud, Anne Hee Hiong Ngu

**Affiliations:** Department of Computer Science, Texas State University, San Marcos, TX 78666, USA; bgu9@txstate.edu (S.T.H.); stg60@txstate.edu (M.D.); nuc4@txstate.edu (A.Y.); tarek_mahmud@txstate.edu (T.M.)

**Keywords:** fall detection, deep learning, LSTM, transformers, wearables

## Abstract

Falls are the second leading cause of unintentional injury deaths worldwide. While numerous wearable fall detection devices incorporating AI models have been developed, none of them are used successfully in a fall detection application running on commodity-based smartwatches in real time. The system misses some falls, and generates an annoying amount of False Positives for practical use. We have investigated and experimented with an LSTM model for fall detection on a smartwatch. Even though the LSTM model has high accuracy during offline testing, the good performance of offline LSTM models cannot be translated to the equivalence of real-time performance. Transformers, on the other hand, can learn long-sequence data and patterns intrinsic to the data due to their self-attention mechanism. This paper compares three variants of LSTM and two variants of Transformer models for learning fall patterns. We trained all models using fall and activity data from three datasets, and the real-time testing of the model was performed using the SmartFall App. Our findings showed that in the offline training, the CNN-LSTM model was better than the Transformer model for all the datasets. However, the Transformer is a preferable choice for deployment in real-time fall detection applications.

## 1. Introduction

Falls are the second leading cause of unintentional injury deaths worldwide. Adults older than 60 suffer the greatest number of fatal falls [1]. The resultant inactivity caused by a fall in older adults often leads to social isolation and increased illnesses associated with inactivity, including infections and deep vein thrombosis. Consequently, a large variety of wearable devices which incorporate fall detection systems have been developed [2,3,4,5].

**Figure 1:**
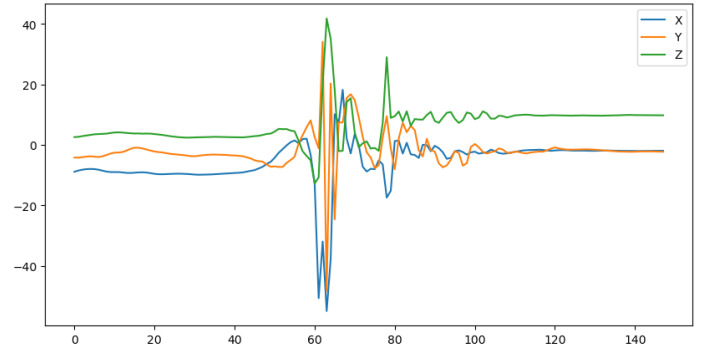
Acceleration from a fall.

One of the main sensors used in fall detection on a smartwatch is an accelerometer, which measures the acceleration of an object. Acceleration is the change in velocity over time, and velocity represents the rate at which an object changes its position. Acceleration data are commonly used in fall detection because a distinct change in acceleration happens when a fall occurs. The clustered spikes in Figure 1 show a unique pattern in the acceleration data during the time when the fall occurs, which means that falls can be identified in acceleration data by that pattern.

Previously, we have developed a watch-based SmartFall App using a basic Long Short-Term Memory Neural Network (LSTM), a Recurrent Neural Network (RNN) with feedback connections, to detect falls based on the above pattern [6,7]. We have deployed this SmartFall system on a commodity-based Huawei smartwatch, which has been trialed by nine senior participants [8]. Despite the system being welcomed by the participants in our trials, it still has several limitations; for example, a sudden hand or wrist movement from some Activities of Daily Living (ADLs) can interfere with the recognition of fall patterns, which resulted in too many False Positives for practical use.

Our existing smartwatch-based fall detection system based on the LSTM model is currently under-performing in real-world testing. As pointed out by [9], there are a few inherent challenges in applying deep learning to wearable devices such as smartwatches. For example, the sensed data could be noisy due to lower Precision sensor readings. The way data are assigned for training may be limiting as well. For example, the LSTM model is not efficient in processing a long sequence of streaming data. Therefore, input data must be segmented into windows. If the chosen window size is too small, important signals might fall outside the range; having the window size too large risks having to process useless input data that is not relevant to a fall (i.e., background noise). We want to investigate whether an alternative model, called the Transformer model [10], can perform better than the LSTM fall detection model. One of the primary merits exhibited by the Transformer architecture in contrast to LSTM is its intrinsic ability to concurrently process data, a characteristic that effectively mitigates the challenges associated with the processing of long sequences. This feature significantly enhances the model’s capacity to handle long input sequences efficiently. Moreover, the self-attention head in the Transformer can selectively focus on different parts of sequential input data, and capture context information that is important for understanding the meaning of the whole sequence which could outperform the LSTM model.

We compared three variants of LSTM models with two variants of Transformer models across three fall datasets. The best LSTM and Transformer model trained offline using the SmartFallMM dataset (data collected in our lab with a smartwatch as one of the sensors) is then converted to TensorFlowLite model and deployed on SmartFall App. We are the first to conduct a real-time evaluation of a TensorFlowLite fall detection model using a real-world SmartFall application. The main contributions of this paper are:

- Evaluate the offline trained best variant of Transformer and LSTM models in real-time on a fall detection application (SmartFall) running on a commodity-based smartwatch running on WearOS.
- Demonstrate that though CNN-LSTM performs better with an F1-Score of 86.7% in offline evaluation, the basic Transformer that has an offline F1-Score of only 82.6% can maintain better performance in real-time testing, making it a more suitable and dependable model for real-world deployment.
- Demonstrate that both variants of Transformers and LSTMs perform better as the size of the dataset increases.
- Demonstrate that the Transformer can maintain better Recall and generate fewer False Positives in real-time testing. This can be ascribed to its sophisticated capacity or self-attention to recognize and comprehend the underlying patterns in the data.

The remainder of this paper is organized as follows. Related works to our research are described in Section 2. In Section 3, we discuss the architecture of the variants of LSTM and Transformer models. In Section 4, the methodology for the experiments is discussed. The computation model is presented in Section 5, while the experimental results are shown and compared in Section 6. In Section 7, we present our conclusions. Finally, in Section 8, we discuss future work.

## 2. Related Work

The early works in fall detection technologies were concentrated on specially built hardware that a person could wear or install in a specific facility [11]. Fall detection devices, in general, try to detect a change in body orientation from upright to lying that occurs immediately after a large negative acceleration to signal a fall. Those early wearable devices are not well-accepted by older adults because of their obtrusiveness and limited mobility. However, modern smartphones and smartwatches now contain more sensors than ever before. Data from those devices can be collected more easily and more accurately with the increases in the computing power of those devices. Smartphones are also widespread and widely used on a daily basis by people of all ages. There has thus been a dramatic increase in the research on wearable-based fall detection and prevention in the last few years. This is highlighted in the survey paper [12]. Smartphone-based fall detection solutions, in general, collect accelerometer, gyroscope, and magnetometer data for fall detection. Among the collected sensor data, the accelerometer is the most widely used. The collected sensor data were analyzed using two broad types of algorithms. The first are threshold-based algorithms, which are less complex and requires less computation power. The second are machine learning-based fall detection solutions. The dynamic of the falls of different people cannot be captured in any rule-based or threshold-based systems. For example, falls from older adults are generally considered as “soft falls”, versus “hard falls” when a person falls from a bike. Moreover, threshold-based algorithms cannot be generalized to the variability of falls and ADLs from people of different heights and weights.

The Support Vector Machine (SVM) learning algorithm has been used for fall detection by scholars in the early days, in [13]. These scholars used a trunk-mounted tri-axial sensor (a specialized hardware) to collect and sense data. They were able to achieve 99.14% accuracy with four features using only high-pass and low-pass accelerometer data. They used a 0.1-second sliding window to record the minimum and maximum directional acceleration in that period for a feature.

Other works in fall detection have focused on using multiple sensors attached to the subject. For instance, sensors can be placed on the lapel, trunk, ankle, pocket, and wrist. These systems typically show marvelous results of 100% accuracy, but lack convenience and portability, and are too computationally intense for a smartphone, due to more data needing to be collected and processed in real-time.

There has been a lot of research work on using Recurrent Neural Networks (RNNs) or Convolutional Neural Networks (CNNs) to detect falls in recent years: in [14], the authors describe an RNN architecture in which an accelerometer signal is fed into two Long Short-Term Memory (LSTM) layers, and the output of these layers is passed through two feed-forward Neural Networks. The second of these networks produces a probability that a fall has occurred. The model is trained and evaluated on the URFD dataset [15], which contains accelerometer data taken from a sensor placed on the pelvis, and produces a 95.71% accuracy. The authors also describe a method to obtain additional training data by performing random rotations on the acceleration signal; training a model with this additional data gives an increased accuracy of 98.57%.

The authors in [16] also proposed an RNN to detect falls using accelerometer data only. The core of their Neural Network architecture consists of a fully connected layer, which processes the raw data, followed by two LSTM layers, and ends with another fully connected layer. The normalization and dropout layers are introduced in their architecture for generalization. The authors train and test their model with the SisFall dataset [17], which contains accelerometer data sampled at 200 Hz collected from a sensor attached to the belt buckle. In order to deal with a large imbalance in training data, of which ADLs form the vast majority, the authors define a weighted-cross entropy loss function, based on the frequency of each class in the data set that they use to train their model. In the end, their model attains a 97.16% accuracy on falls and a 94.14% accuracy on ADLs. However, it is unrealistic to expect a commodity-based wearable device to sample at 200 Hz in real time because of battery constraints. Moreover, none of these RNN-based models have been tested or trialed in the real world.

Our earlier work [18] compared traditional machine learning (SVM, Naive Bayes) techniques with deep learning (DL) model, in particular, the Recurrent Neural Network (RNN), for fall detection using only acceleration data captured through a commodity-based Microsoft Band wrist-worn watch, and concluded that the DL model has better fall detection performance. The best model we obtained using RNN on our SmartFall dataset [19] collected with a Microsoft MSBAND watch has an overall accuracy of 86% in the offline test. Because of its placement on the wrist, a smartwatch will naturally show more fluctuation in its measurements than a sensor placed on the pelvis or belt buckle. In addition, data sensed by a commodity watch is noisier than specialized equipment. The computational power of a smartwatch is constrained and, thus, we adopted a basic LSTM model with only two dense layers. Our LSTM model is one of the few models that have been trialed in the real world [8]. We have also benchmarked a 1D-CNN against stacked/ensemble LSTM models in [6], and found the stacked LSTM to perform better. This demonstrated that despite being constrained with a smaller dataset, a deep-learning-based approach can learn the fall patterns better than traditional machine learning techniques.

In [20], a hybrid of CNN and LSTM architecture, called ConvLSTM, is proposed. ConvLSTM can predict falls with a Recall of 96% and a Precision of 98.69% using the SisFall dataset with 5-fold cross-validation. The CNN layer acts as a feature extractor, and provides an abstract representation of the input sensor data in feature maps. In other words, the CNN layer captures the spatial relationship in the data, while the LSTM layer captures the long-term temporal relationship. They demonstrated that ConvLSTM outperformed models that are solely CNN or LSTM. In [21], a DeepConvLSTM was presented for recognizing two families of human activities (periodic motion activities, such as walking and bicycling, and gestures such as drinking water from a cup) on the Skoda and OPPORTUNITY datasets. The DeepConvLSTM achieved the F1-Score of 93% on the OPPORTUNITY dataset. The resulting model has large parameters, and data are sensed from multiple locations of the body, which makes it impractical for real-world use. DeepConvLSTM has also shown that for sequential data a combination of recurrent LSTM units and Convolutional Neural Networks can outperform CNN models.

Transformer has recently achieved a state-of-the-art in natural language processing (NLP) tasks. The popular ChatGPT tool uses Transformer blocks in its architecture. Transformer, when used in natural language processing, has been shown to outperform older approaches for sentence classification, which made use of conventional RNNs/LSTMs  [10]. Transformers can efficiently process long sequences, and support parallel computing with fast computation. Transformers have recently gained popularity for fall detection. For example, in [22], they demonstrated that using the accelerometer signals of a waist-worn Inertial Measurement Unit (IMU) can gain a noticeable gain in accuracy of the model of 95.7% using the SisFall dataset. In the Edge Impluse Studio Project [23], a Fall Detection model trained with the Transformer model using the SisFall dataset was deployed on a resource-constrained Arduinio microcontroller with an ADXL345 accelerometer sensor. The model accuracy for fall is around 88%. None of the trained Transformer models have been tested in the real world.

In the Patch-Transformer Network [24] for fall detection, the network includes a convolution layer, a Transformer encoding layer, a global average pooling layer, and a linear classification layer. The convolution layer is used to extract local features. Global features of falls are learned through the Multi-Head Self-Attention mechanism in the Transformer encoding layer. The final classification is provided by the linear layer. The accuracy result obtained using the SisFall dataset for training is 99.86%, with a detection time of 0.004 s. They demonstrated that an accurate Transformer model with a low number of training parameters and model complexity can be obtained with three attention heads and a maximum of six encoding layers. They demonstrated that there is an advantage in adding a CNN layer to a Transformer for better detection in fall patterns.

There is no systematic study that compares fall detection models trained using variants of LSTM and Transformer. In [25], the authors compared LSTM with self-attention to Transformer, and concluded that an LSTM with self-attention gave better model accuracy than a Transformer across four public time series datasets (only one of the datasets is related to falls). However, the trained model is not evaluated in real-time on any wearable device. It is reported in [26] that only 7.1% of fall detection projects performed real-world testing on their models. In particular, we are interested in evaluating whether there is a distinct advantage of using a Transformer over an LSTM in obtaining better model accuracy and acceptable real-time performance of the model on a wearable device. For fall detection technologies to be adopted by older adults, the trained offline model must be tested and verified in the real world.

## 3. Architecture of Models

In the literature, there is no consensus on what is the best LSTM or Transformer architecture for fall detection using only accelerometer data from a wristwatch. There is also no universal standard fall dataset that can be used for comparison. We choose to use three variants of the LSTM model. The first one is the basic LSTM model, which has been deployed by us successfully in the SmartFall App running on a Huawei watch; the second is a hybrid CNN and LSTM model, which has shown good performance by several scholars; and the last one is LSTM with self-attention. The latter has been compared with the Transformer model in [25], and demonstrated better performance over the Transformer. For the Transformer, we adopted a vanilla Transformer used in our SmartFall App, and also a ConFormer from [27].

### 3.1. LSTM

The basic LSTM architecture we used contains an input layer, an LSTM layer, a dense layer, a batch normalization, and an output layer. The input layer contains three nodes for the raw data; the accelerometer *x, y, z* vectors are provided with a variable input shape of (*W*, 3, 64), where *W* denotes window size, 3 signifies the *x, y, z* values of accelerometer data, and 64 is the batch size used. It then feeds through a recurrent LSTM layer and a fully connected dense layer. The output is a sigmoid layer that outputs a predicted probability that a fall has occurred.

**Figure 2:**
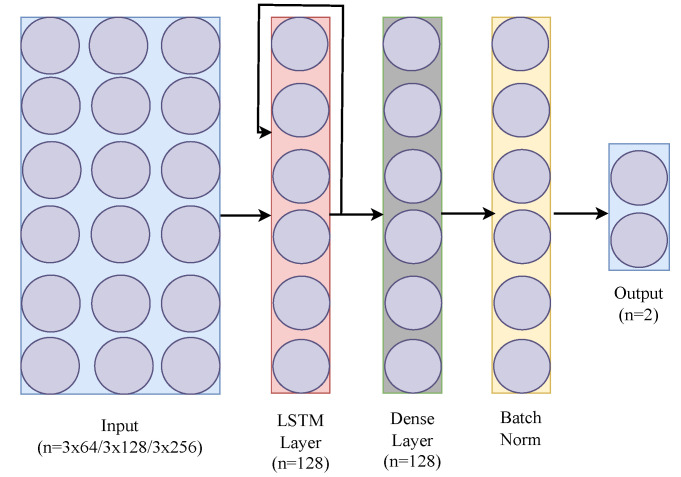
Basic LSTM architecture.

The Binary Cross Entropy (BCE) with ADAM optimizer is used. RNNs are traditionally trained with backpropagation through time (BPTT), so it is necessary to specify how many steps *n* in the past the network should be trained on. This defines the size of the window *W* of data points that must be fed to the model at each data sample selection. Figure 2 shows the basic LSTM that we have used in our study.

**Figure 3:**
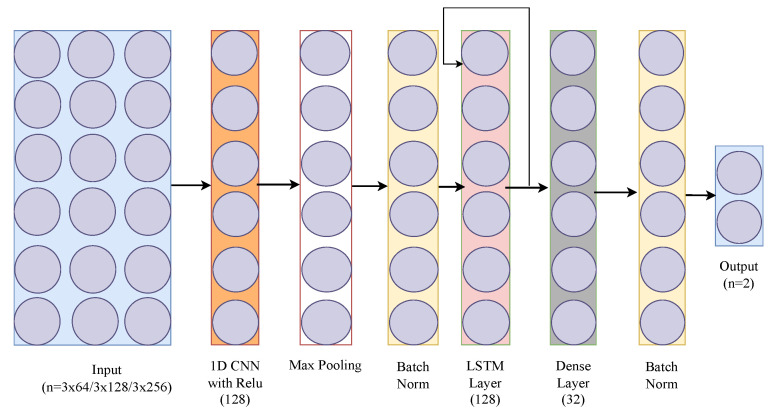
CNN-LSTM architecture.

The CNN-LSTM we used integrates two fundamental Neural Network components: Convolutional Neural Networks (CNNs) and Long Short-Term Memory (LSTM) networks. It starts with a 1D convolutional layer with a ReLU activation function, followed by max-pooling to capture local patterns in the input sequence. This combination is repeated for increased feature extraction. A subsequent LSTM layer is introduced to capture long-term dependencies in the data. Dense layers with a ReLU activation follow. Batch normalization is applied throughout the model to enhance training stability. The final output layer employs a sigmoid activation function for binary classification. The model is compiled using BCE and an Adam optimizer. Figure 3 shows the CNN-LSTM that we have used in our study.

**Figure 4:**
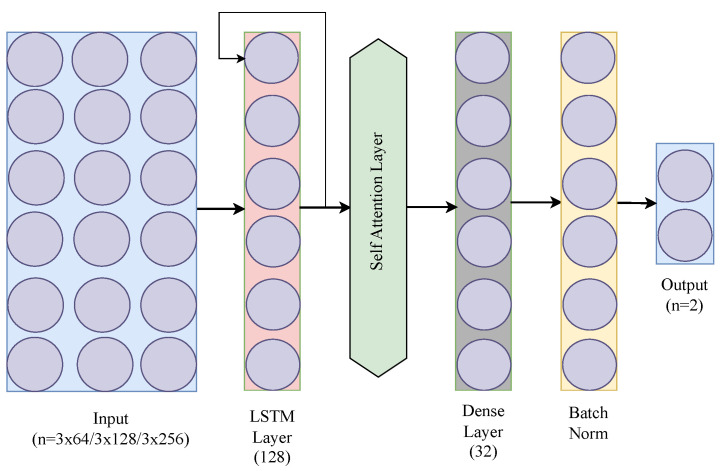
LSTM with self-attention architecture.

We implemented another variant of the LSTM model, called LSTM w/self-attention, where a self-attention layer is incorporated into the basic LSTM implementation to enhance its ability to process time series data. Self-attention is a mechanism that allows the model to weigh the importance of different parts of the input data. It helps the model to focus on the important time points of the input sequence for making predictions. Here, the output of the LSTM layer is passed to a self-attention layer. This means that the sequential output of the LSTM is further processed by the attention mechanism. This addition enriches the model’s capacity to identify crucial patterns in data while maintaining its lightweight nature for wearable devices. The model, still trained using backpropagation through time (BPTT), benefits from this advanced feature, offering a more nuanced understanding of temporal dynamics without compromising efficiency. Figure 4 presents the architecture of LSTM with self-attention that we have used. In these three Figure 2, Figure 3, Figure 4 we have used different colors to distinguish the layers of each architecture. For instance, the LSTM layers are represented by the same color across all three figures, while the dense layer is assigned a unique color to differentiate it. This approach has been applied consistently to all the other layers as well.

**Figure 5:**
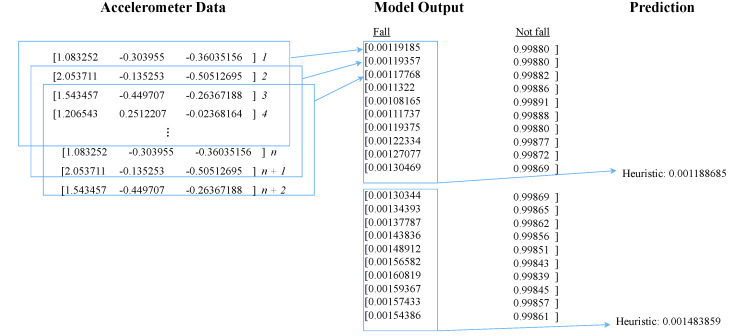
Prediction scheme for LSTM model.

Fall prediction using all three variants of LSTM-based architecture is made on a sliding window of data that is *W* data points in length. Each prediction outputs a probability of fall between 0 and 1. Figure 5 depicts the prediction schematic. The model prediction begins once the number of sensor data points acquired is equal to the number of the configured window size *W*. Every model prediction thereafter will only require additional data points, which is the step size *S*. That means the consecutive windows have a W−S time step overlap. However, before producing a final prediction, we generate a heuristic value based on the probabilities produced by the average of 20 consecutive model predictions. This value is derived from experimentation, with values ranging from 1 to 20 with five increments. In essence, we compute the average value of 20 consecutive probabilities and compare this with a pre-defined threshold value. During the training and validation of the model, a threshold of 0.5 yields the optimal results, and is used in the SmartFall App. If the average probability exceeds this threshold, then it is considered a fall prediction. This helps to prevent isolated positive model predictions from triggering a False Positive.

### 3.2. Transformer

**Figure 6:**
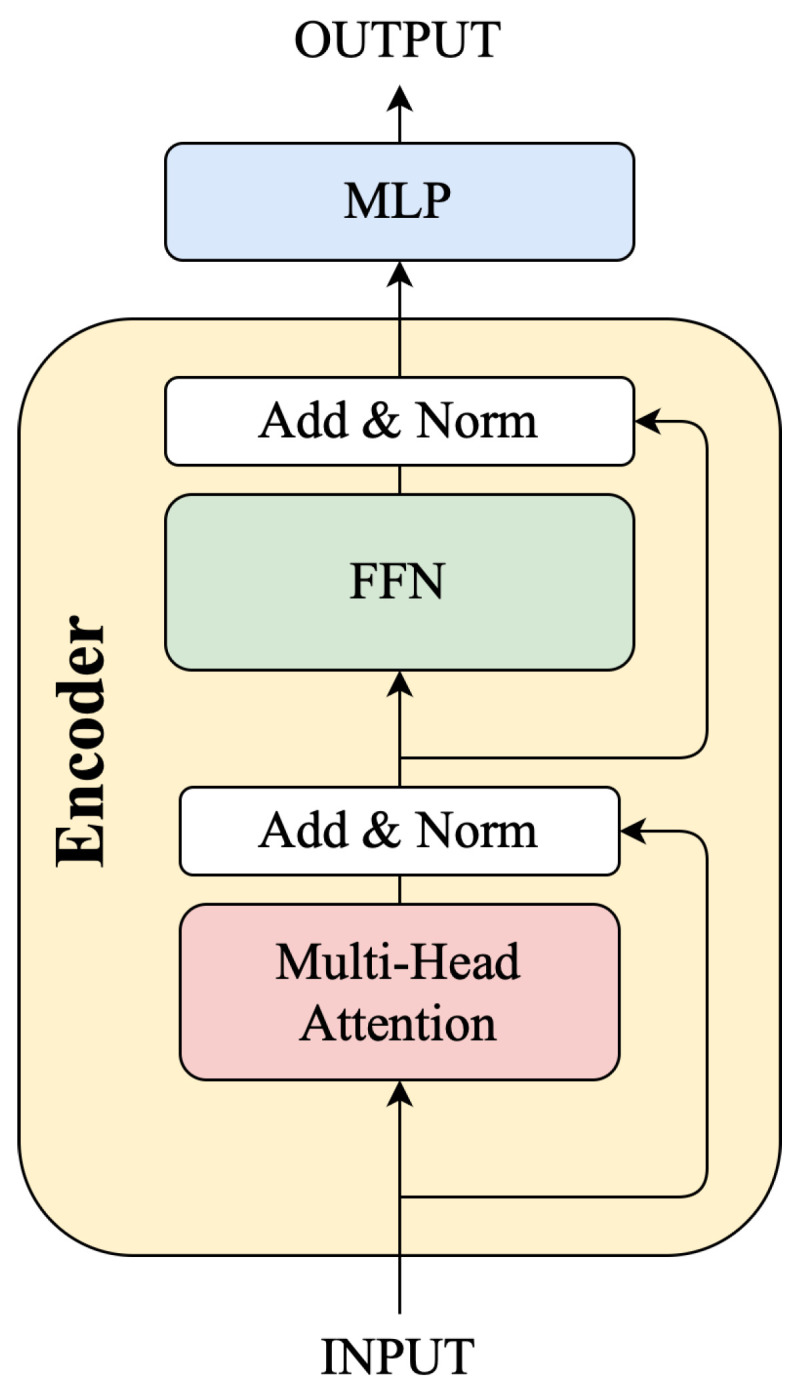
Transformer architecture.

We implemented the Transformer architecture described in [10], which has an encoder–decoder structure. We chose the encoder part for fall detection, since it has been shown in [28] that the Transformer can achieve high accuracy for human activity recognition. This architecture, as shown in Figure 6, has four encoder layers, and four attention-heads in each layer to balance the model size and performance metrics when deployed on power- and computation-constrained wearable devices. The original Transformer also has a decoder part that generates a new sequence in machine translation. We do not need any decoder layers for fall detection, as we do not need to re-construct any new sequences. The attention mechanism is essential in the Transformer model, with multiple attention heads seeking different relevance definitions or correlations [28]. Multi-Head Attention (MHA) involves mapping queries and key–value pairs to an output. In a single attention head, scaled dot products of queries(Q) and keys (K) are calculated, followed by softmax normalization, which yields weights that are multiplied by values (V), as shown in Equation (1). The output from multiple attention heads is then concatenated and projected to obtain the final output, as shown in Equation (2). The Multi-Head Attention is followed by Layer Normalization and a skip connection with input before the Multi-Attention.

Layer normalization is a technique used in the Transformer architecture to normalize the activation of each layer independently, stabilizing training by addressing issues like vanishing gradients and internal co-variate shifts. It improves gradient flow, accelerates convergence, and reduces sensitivity to hyperparameters. This contributes to the model’s effective training and enhanced performance.

The last layer of the Transformer encoder is the feed-forward network/module (FFN). It independently processes each position in the input sequence using Equation (3). This step enhances the model’s ability to capture complex relationships within the data. The output of the last encoder layer is fed into an MLP layer consisting of three linear layers with 8, 16, and 1 neurons, respectively, to classify falls and ADLs. The sigmoid function was used as an activation function of the output layer to yield a probability between 0 and 1.

(1) $$\mathrm{Attention}\left(Q,K,V\right)=\mathrm{softmax}\left(\frac{{\mathrm{QK}}^{T}}{\sqrt{d_{k}}}\right)V$$

(2) $$\mathrm{MHA}\left(Q,K,V\right)=\mathrm{Concat}\left({\mathrm{head}}_{1},\ldots ,{\mathrm{head}}_{h}\right)\cdot W^{O}$$

(3) $$\mathrm{FFN}\left(x\right)=\max\left(0,x\cdot W_{1}+b_{1}\right)\cdot W_{2}+b_{2}$$

**Figure 7:**
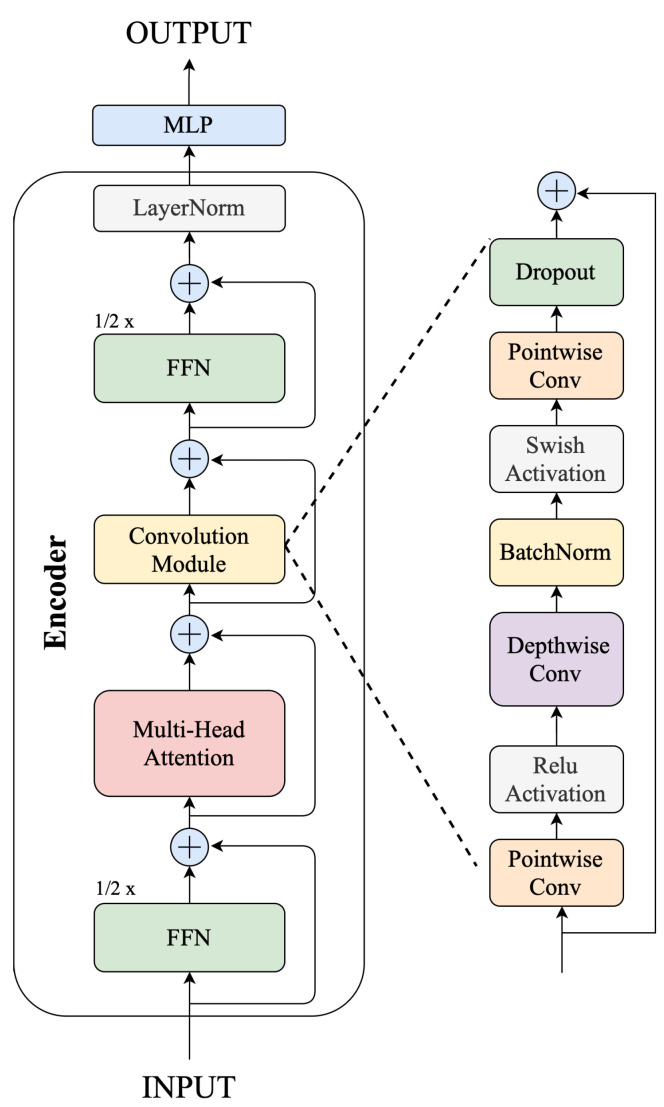
ConvTransformer architecture.

The ConvTransformer, as shown in Figure 7, was inspired by [27]. The encoder layer of it has a sandwich-like structure, where two half-feed forward networks (FFNs) are sandwiched between the Multi-Head Self-Attention module and the Convolution module. The sandwich-like architecture was inspired by Macaron-Net [29]. Starting from scratch, the convolution module combines a ReLU activation with a pointwise convolution. Then, there is only one 1-dimensional depthwise convolution layer used with swish activation. To make training deep models easier, batch normalization is added right after the depthwise convolution process. The sandwich-like structure and convolution module differentiate ConFormer and basic Transformer. We refer to this architecture as ConvTransformer in this paper.

## 4. Methodology

### 4.1. Dataset

We evaluated variants of LSTM and Transformer on three datasets: UniMib [30], K-fall [31], and SmartFallMM (https://anonymous.4open.science/r/smartfallmm-4588; data collected in our lab; accessed on September 23, 2024). Falls are rare events, and it is very labor-intensive to collect large amounts of fall data. The largest public dataset we found that has a practical sampling rate for wearable devices, types of falls, and ADLs is the K-fall dataset.

UniMib is a human activity recognition dataset of acceleration data collected from smartphones. The data were collected from 30 subjects, with ages ranging from 18 to 60 years. The participants performed 9 types of Activities of Daily Living (ADLs) and 8 types of falls. The data were collected at a sampling rate of 50 Hz. The participant puts phones in the left and right pockets of trousers, and hand clapping is used to signal the beginning and end of a fall. We processed this dataset, and have all nine types of ADLs labeled as “NonFall”. We only retained the five common fall types that we used in our SmartFallMM fall dataset for ease of comparison. The final number of falls is 710 and ADLs is 486.

The K-fall dataset is built for pre-fall, fall, and post-fall detection. In total, 21 types of Activities of Daily Living and 15 types of simulated falls were performed by 32 young and healthy participants. A nine-axis inertial sensor was attached to the participant’s lower back to collect the accelerometer, gyroscopes, and magnetometer data. We did not filter out any type of falls, so that a reasonably large dataset could be used. In total, K-fall has 5075 motion files, including 2729 ADLs and 2346 falls.

**Figure 8:**
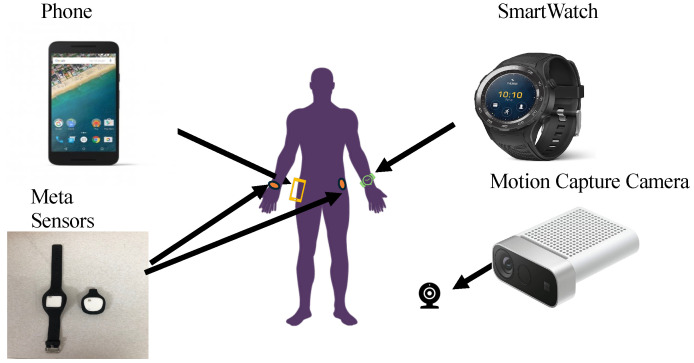
Sensors placement on the participants.

SmartFallMM is a multimodal dataset focused on falls and ADLs data, gathered from 16 student participants (11 male and 5 female) with a median age of 23, and 26 older participants (12 male, 14 female) with a median age of 65.5. Our multimodal data collection was approved by IRB 9461 at Texas State University, and was the first multi-modal dataset that has both older and younger adults’ data. We only asked student participants to perform falls on an air mattress. All participants needed to sign consent forms before their data could be collected. Two modalities were collected using four types of devices. We collected skeleton data using Azure Kinect cameras, time-series data such as accelerometer data, and gyroscope data using three inertial sensors (i.e., meta sensor, Huawei Smartwatch, and Nexus phone). Meta sensor was developed by MBIENTLAB in San Jose, CA 95124, USA (mbientlab.com); accessed on September 23, 2024. The participant wears the Huawei watch on the left wrist and puts the Nexus smartphone on the right hip inside a harness. A wrist meta sensor was placed on the right wrist of the participant, and the hip meta sensor was placed on the left hip of the participant (clipped onto the belt). This setup allowed us to collect data from four important joints of the human body when a person moves. Figure 8 shows the positions and the types of sensors used for the data collection.

For training and comparative purposes of LSTM and Transformer models for fall detection, we used only the accelerometer data captured at 32 Hz from the Huawei watch worn on the left wrist of the 16 student participants, because it had both fall and ADL data. We did not and could not collect fall data from older adults. Five types of falls and eight types of ADL activities were collected. The subset of the SmartFallMM dataset we used had a total of 560 ADLs and 400 Falls. Table 1 gives a summary of the three datasets used for our experiments.   

### 4.2. Input Data Processing

We used the sliding window technique to divide the input data into a series of overlapping windows. Algorithm 1 shows the technique used for the sliding window. The window size *W* in the equation below indicates how many data points the model sees at each prediction, and the step parameter dictates how far the window moves down during each iteration of sample selection. We utilized a step size of 10, which allows ten new data points to be added to every new window created. We have experimented with different step sizes, ranging from 1 to 50, and found that using a step size of 10 captures more meaningful information when defining training instances. We experimented with different window sizes of 64, 128, and 256 in our study.
**Algorithm 1** Sliding window algorithm1:**Input:** Input dataframe df, window size *W*, step size step2:**Output:** Output dataset of all windows windows3:Initialize an empty array windows4:**for**i←0:step:lengthofactivity**do**5:   Set currentWindow as the subarray of df from index *i* to i+W−16:   Append currentWindow to windows7:**end for**

## 5. The Computational Model

### 5.1. Hyperparameters

We train the models using TensorFlow on a Dell Precision 7820 Tower with 256 GB RAM and one GeForce GTX 1080 GPU by Dell EMC at Sugarland, Texas.

We tried to keep similar hyperparameters for both variants of models as much as possible. However, due to the salient differences in their architecture, some differences were unavoidable. The best hyperparameters used for the variants of Transformer and LSTM are listed in Table 2. The “*” on Transformer and LSTM in Table 2 are used to refer to the different variants of the two type of models.

### 5.2. Training and Evaluation

As our dataset is not very large, we employed a Leave-One-Out strategy, wherein the entire set of activities associated with a particular individual is reserved exclusively for testing. This strategic maneuver ensures that the model remains entirely unexposed to any data emanating from a given participant during the training phase. By adopting such an approach, we effectively mitigate the risk of data leakage, thereby having a more reliable and robust assessment of model generalization on unseen data.

### 5.3. Evaluation Metric

To evaluate and compare the performance of Transformer and LSTM, we look at the *F1* score, *Precision*, *Recall* and *Accuracy*. These metrics are defined as:

$$Precision=\frac{TP}{TP+FP}$$

$$Recall=\frac{TP}{TP+FN}$$

$$F1\_Score=\frac{2*Recall*Precision}{Recall+Precision}$$

$$Accuracy=\frac{TP+TN}{TP+TN+FP+FN}$$

True Positive (*TP*) occurs when the model correctly predicts a positive instance, such as accurately detecting a fall. True Negative (*TN*) is when the model correctly predicts a negative instance (ADL, in our case). False Positive is the case when the model erroneously predicts a negative data sample as positive, and False Negative is the opposite case. While Precision assesses the accuracy of positive prediction, Recall quantifies the correct identification of real positives. The *F1-Score* is a harmonic mean of precision and recall used to provide a balanced measure of model performance.

### 5.4. Model Evaluation Method

The experiment explores two forms of model evaluation. The traditional offline machine learning model evaluation and the real-time evaluation with the SmartFall App, which is a fall detection application developed in our lab. In the offline model evaluation phase, a model is trained on a training dataset, tested on a validation set to obtain the best hyperparameters, and the best-validated model is used on the test data.

We selected only the two best Transformer and LSTM models trained on the SmartFallMM dataset for real-time evaluation. The model trained with a window size of 128 has the highest *F1-Score* for both the Transformer and LSTM, and was chosen for real-time evaluation. The models trained with UniMib and K-fall cannot be used for real-time testing by the SmartFall App [32] because the data were not collected using a smartwatch at a wrist position.

**Figure 9:**
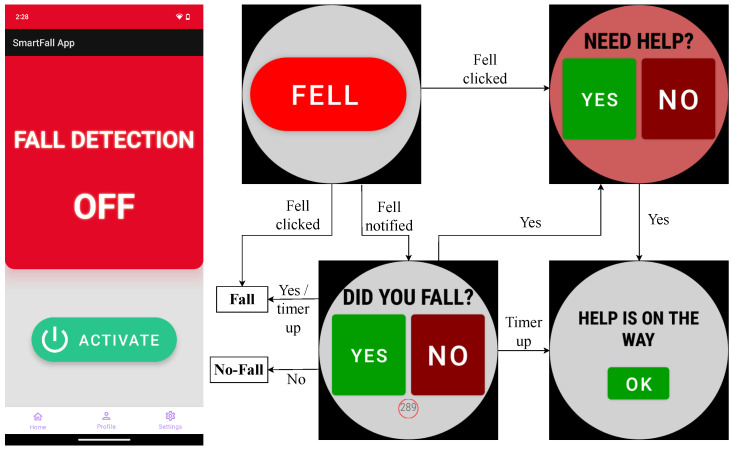
User interface of SmartFall app for real-time evaluation.

Figure 9 shows the phone and watch’s UI in the SmartFall application. We followed the best practices advocated in the literature for the design of the UI for older adults. The three main principles we adopted were a strict color scheme with high contrast, legible and big fonts, simple descriptions of the system to engage them to use it. The background of the “NEED HELP?” screen is changed to red color to distinguish it from the “DID YOU FALL?” screen. The users must react correctly to these two screens. After pairing the phone and the watch, as soon as the user presses “activate” on the phone’s UI, the sensor on the watch will sense the accelerometer data from the watch continuously. Model predictions (i.e., predictions produced by the neural architecture) begin once the number of sensor data points acquired is equal to the defined window size.

We recruited three student participants for the real-time evaluation of LSTM and Transformer models at our lab. Our real-time evaluation of the model was also covered under the IRB 9461 at Texas State University. We asked each student participant to sign a consent form. The consent form gave the student participant information on why this research study was being performed, and described what they would need to do to participate, as well as any known risks, inconveniences, or discomforts that they may have had while participating. Each student participant was asked to wear the watch on the left wrist and perform the five types of falls on a 12-inch high queen-size air mattress. The same participant was also asked to perform eight prescribed lists of ADLs.

## 6. Results

### 6.1. Results of Traditional Offline Model Evaluation

Table 3 showed the performance of five different machine learning models across three datasets: SmartFallMM, UniMib, and K-fall, with varying window sizes of 64, 128, and 256. The performance metrics used for comparison were Precision, Recall, and F1-Score.

As shown in Table 3, on the SmartFallMM dataset, the models demonstrated varied performance across different window sizes. The basic Transformer model exhibited an increase of 8.6% in F1-Score with the window size increasing from 64 to 128. The basic Transformer model has the highest F1-Score of 82.6% with a window size of 128.

The basic LSTM showed a relatively lower F1-Score across all window sizes, indicating that simple recurrent structures might not be sufficient for the complexity of the task. The CNN-LSTM performed at higher F1-Scores. The best-performing variant of the LSTM model is CNN-LSTM, with a score of 87.6% at a window size of 128. A 7.2% improvement in the F1-Score for the LSTM with attention for windows size of 256, compared to the basic LSTM, indicates that self-attention effectively captures relationships between different timesteps.

Results on the UniMib dataset were less consistent. The basic Transformer model improved by 3.6% when going from a window size of 64 to 128. The ConvTransformer showed its highest F1-Score of 86.6% at the mid-window size of 128, suggesting a balance between input sequence length and the influence of spatial model capability. The LSTM models, particularly the CNN-LSTM and LSTM with self-attention, again demonstrated strong performance, with the CNN-LSTM performing at the highest F1-Score (93.7%) with the smallest window size of 64. This might suggest that when the dataset is small, the influence of the convolutional layer is stronger.

For the K-fall dataset, the trend shifted with the ConvTransformer achieving the highest F1-Score of 94.0% at the largest window size of 256, surpassing the basic Transformer model by 4% at that window size. Moreover, the Recall and Precision are both above 90%. A similar trend is observed in all the variants of LSTM models. The better performance of models with a larger window size in K-fall can be attributed to the higher sampling rate of K-fall data. K-fall has a sampling rate of 100 Hz, which is double that of UniMib with 50 Hz and SmartFallMM with 32 Hz. As the K-fall had more data points per second, a bigger window size can accommodate the whole duration of the fall sequence better.

K-fall is the largest dataset we used, and our experimental results thus indicate that there is a definite increase in F1-Score by training with a bigger dataset across all the variants of Transformer and LSTM. The model trained with the bigger dataset also presented the most balanced and consistently high Precision and Recall. For the offline test, the CNN-LSTM, which can learn both the spatial and temporal nature of the data, performs better than both the basic Transformer and ConvTransformer. We believe this might be because the Transformers do not have any inductive bias for temporal information, and ConvTransformer only has bias for spatial information.

### 6.2. Results of Real-Time Evaluation

For the real-time evaluation of the models, we choose the best-performing offline model of Transformer and LSTM trained using the SmartFallMM dataset with a window size of 128, as shown in Table 3. We use the terms Transformer and basic Transformer synonymously here, for ease of reference. The performance of each model for three young adults is shown in Table 4. Each participants performed each activity five times. The outcome is recorded as “Yes” if a True Positive is detected. The Transformer model consistently outperformed the CNN-LSTM model for all three participants. For the Participants 1 and 2, the Transformer outperforms CNN-LSTM across all evaluation metrics. Although, for Participant 3, the CNN-LSTM performs better in terms of F1-Score by 2.2%, it misses some falls. In addition, the average F1-Score (77.1%) of the Transformer in real-world test is closer to the F1-Score (82.6%) of the Transformer model trained offline. However, that is not the case for the CNN-LSTM model, which has an offline F1-Score of 87.6%, but only achieved a 70.2% F1-Score in a real-world test. This indicates that only the Transformer model can transfer the offline model result to the real world.

To analyze the model’s performance in more detail, we examined the individual fall activities’ detection outcomes, as displayed in Table 5. By examining the table, it becomes evident that certain falls are readily identifiable, as both models exhibit comparable accuracy in these cases. The main difference between the Transformer and LSTM is imminent in case of detecting the Left and Right falls. For instance, both models detected Front, Back, and Rotate falls. For all three participants, the Transformer does not miss any of the falls, whereas CNN-LSTM missed one Left fall and two Right falls for Participant 3.

The three participants also performed eight ADL activities with a repetition of five of each activity, which amounts to 40 activities in total for each participant. For the Transformer, the number of False Positives for Participant 1 is 13/40, Participant 2 is 9/40, and Participant 3 is 23/40. For the CNN-LSTM model, the False Positives for Participant 1 is 11/40, Participant 2 is 20/40, and Participant 3 is 15/40. This again indicates that the Transformer can classify ADLs better than CNN-LSTM, which is reflected in the lower Precision scores of CNN-LSTM across the three participants.

The low F1-Score obtained when evaluating the CNN-LSTM model in real-time scenarios indicates that the model struggles to effectively apply its learned capabilities to the real world. Though a CNN-LSTM model with a high 87.6% F1-Score was deployed on the SmartFall App for real-time evaluation, none of the three participants achieved an F1-Score close to that. The highest achieved F1-Score was 70.9% by Participant 1. In contrast, a Transformer model can maintain almost the same high level of F1-Score in real-time circumstances. This suggests that the Transformer model inherits the capability of successfully putting into practice what it has learned and adapted to the dynamic nature of the real-time environment, demonstrating its robustness and reliability for deploying to edge devices.

## 7. Conclusions

We assessed the performance of five deep learning models’ performance on three fall datasets, and evaluated the real-time performance of the best model trained with SmartFallMM data from the variants of LSTM and the Transformer using our SmartFall App. The real-time evaluation confirmed that the Transformer outperforms LSTM in dynamic real-world scenarios. To the best of our knowledge, we are the first to perform comparative studies involving real-time testing of deep learning-based fall detection models on a smartwatch. The only other real-world test of a fall detection app on a smartwatch was with a model using a threshold-based algorithm [33], which achieved a Recall of 77%. This means a threshold algorithm missed 23% of falls in the real-world test, while a deep learning-based model has almost 100% Recall, based on our real-world evaluation. The real-world test is performed using Huawei watch, but our system is not restricted to the Huawei brand of watch. We have used TicWatch in other real-world evaluation studies. Any smartwatch that runs WearOS can be used.

Our findings showed that in the offline training, the CNN-LSTM model was better than the basic Transformer model for all the datasets and all the windows in F1-Scores. This is due to the joint spatial and temporal inductive bias on the CNN-LSTM. In Transformers, due to the lack of inductive biases, they need a lot of data to gain optimal performance. This is observable in the K-fall dataset, as the Transformer achieved an F1-Score of 94.0% for a window size of 256. The Transformer might need an even larger dataset to outperform the CNN-LSTM.

For the real-world test, CNN-LSTM produces a higher number of False Positives than Transformers showing that Transformers can learn complex patterns better due to exploring the relationships in data via the attention heads. The drop in F1-Score is 5.5% for Transformer in the real-time test which is much smaller than CNN-LSTM’s 17.4% which makes the Transformer a more reliable model.

When running the trained model on an edge device like an Android phone, there is a need to convert the trained TensorFlow model to a TensorFlow Lite version. As TensorFlow Lite is a compressed version of the original model, there will be a reduction in model accuracy. This is confirmed by the fact that the F1-Scores from all three participants were lower in the real-time test across both types of models.

Based on our experiments comparing the Transformer and LSTM, our findings suggest that the Transformer may be a preferable choice for deployment in real-time fall detection applications. This comparison could offer valuable insights to future researchers aiming to transition their offline deep learning models into real-world testing. In scenarios like fall detection, it is crucial to consider the feasibility of implementing such models in practical applications, making this a significant consideration. This study also demonstrated that models trained from data collected from a wrist position performed worse than data collected from the hips and lower back. This indicates the challenges of using only the wrist data for fall detection.

The metric we used for comparison is restricted to model accuracy such as Precision, Recall, and F1-Score. The power consumption and inference time are not within the scope of this study. Interested readers can refer to our recent paper “An Empirical Study on AI-Powered Edge Computing Architectures for Real-Time IoT Applications” [32] regarding the impact of software architecture on the power consumption, inference latency, and model accuracy.

## 8. Future Work

Although the Transformer model outperforms CNN-LSTM in terms of our assessment scores in the real-world test, the studied model has been trained so far on only a small-scale dataset, and there are still far too many False Positives. To the best of our knowledge, a larger dataset than SmartFallMM collected using a smartwatch as the sensor is not publicly available at the moment. Since Transformers are known to perform well with bigger datasets, one of our immediate future goals is to increase the size of our dataset by leveraging techniques such as data augmentation, generative models, or extracting from videos of people falling to augment our dataset [34].

Our real-world test is conducted with three participants. To further validate our conclusion that the Transformer is preferable for real-world deployment, we will recruit 10 more participants to validate the rate of True Positives and False Positives of the model this year. Our longer-term goal is to leverage SmartFallMM, the multimodal dataset we collected for multi-modal learning, and to create a fall detection model that can leverage this dataset, consisting of skeleton and accelerometer data during training, and can make inferences only using accelerometer data from the wrist via the knowledge distillation method.

## Figures and Tables

**Table 1:** Summary of the datasets used.

	SmartFallMM	UniMib	K-Fall
Number of ADLs	560	710	2729
Number of Fall	400	486	2346
Number of Subjects	16	30	32
Sampling Frequency	32 Hz	50 Hz	100 Hz
Type of devices	Kinect cameras, phone, watch, meta sensors	Phone	9 axis IMU
Device placement	Left and right wrists and hips	Left and right pockets of pants	Lower back

**Table 2:** Hyperparameters.

Names	Models	
	Transformer *	LSTM *
Learning Rate	0.001	0.001
Epochs	100	100
Batch Size	64	64
Optimizer	Adam	Adam
Loss Function	BCE	BCE
Encoder	4	-
MHA Heads	4	-
MHA Dim	128	-
MLP Dimension	16	128

**Table 3:** Offline evaluation with three datasets. The best-performing model for each dataset is highlighted in bold.

Dataset	Window Size	Metrics	Transformers		LSTM		
			Transformers	ConvTransformers	LSTM	CNN-LSTM	LSTM w/Self-Attn
SmartFallMM	64	Precision	84.9	64.1	64.8	74.7	71.3
		Recall	65.8	72.3	77.2	89.4	82.1
		F1-Score	74.0	67.9	71.2	81.3	76.3
	128	Precision	88.9	74.4	76.5	83.2	76.5
		Recall	77.0	83.1	83.2	92.5	82.9
		F1-Score	**82.6**	78.5	79.7	**87.6**	79.6
	256	Precision	78.3	72.9	64.3	83.5	75.2
		Recall	84.0	89.0	76.8	79.2	79.6
		F1-Score	81.1	80.8	70.1	81.3	77.3
UniMib	64	Precision	79.1	78.4	84.1	94.4	85.0
		Recall	75.5	80.5	89.1	93.6	89.3
		F1-Score	77.2	79.4	86.1	**93.7**	87.1
	128	Precision	84.8	85.6	71.3	83.1	85.8
		Recall	77.2	87.7	88.4	91.2	93.5
		F1-Score	80.8	**86.6**	79.1	87.5	89.4
	256	Precision	70.1	79.0	61.3	85.4	81.1
		Recall	83.9	85.0	81.2	96.1	86.1
		F1-Score	76.4	81.9	70.2	90.3	83.6
K-fall	64	Precision	82.6	72.5	82.4	83.5	78.8
		Recall	84.9	86.3	78.7	81.5	83.9
		F1-Score	83.8	78.8	80.1	82.2	80.1
	128	Precision	84.8	81.1	89.1	85.4	81.0
		Recall	86.8	89.0	85.6	89.1	89.2
		F1-Score	85.8	84.9	87.3	87.4	84.3
	256	Precision	88.5	93.4	95.3	93.1	91.5
		Recall	93.5	95.7	89.6	96.3	93.1
		F1-Score	90.9	**94.0**	92.2	**95.5**	92.3

**Table 4:** Real-time evaluation with SmartFallMM.

	Metrics	Transformer	CNN-LSTM
Participant 1	Precision	65.7	53.1
	Recall	100	100
	F1-Score	78.7	69.2
	Accuracy	80.0	66.1
Participant 2	Precision	73.5	55.5
	Recall	100	100
	F1-Score	84.3	70.9
	Accuracy	86.1	69.2
Participant 3	Precision	52.0	59.4
	Recall	100	88.0
	F1-Score	68.4	70.6
	Accuracy	64.6	76.9
Average	Precision	63.7	56.0
	Recall	100	96.0
	F1-Score	77.1	70.2
	Accuracy	76.9	70.7

**Table 5:** Real-time evaluation using SmartFall app.

		Transformer					CNN-LSTM				
		Trial 1	Trial 2	Trial 3	Trial 4	Trial 5	Trial 1	Trial 2	Trial 3	Trial 4	Trial 5
Participant 1	Front Fall	Yes	Yes	Yes	Yes	Yes	Yes	Yes	Yes	Yes	Yes
	Back Fall	Yes	Yes	Yes	Yes	Yes	Yes	Yes	Yes	Yes	Yes
	Left Fall	Yes	Yes	Yes	Yes	Yes	Yes	Yes	Yes	Yes	Yes
	Right Fall	Yes	Yes	Yes	Yes	Yes	Yes	Yes	Yes	Yes	Yes
	Rotate Fall	Yes	Yes	Yes	Yes	Yes	Yes	Yes	Yes	Yes	Yes
Participant 2	Front Fall	Yes	Yes	Yes	Yes	Yes	Yes	Yes	Yes	Yes	Yes
	Back Fall	Yes	Yes	Yes	Yes	Yes	Yes	Yes	Yes	Yes	Yes
	Left Fall	Yes	Yes	Yes	Yes	Yes	Yes	Yes	Yes	Yes	Yes
	Right Fall	Yes	Yes	Yes	Yes	Yes	Yes	Yes	Yes	Yes	Yes
	Rotate Fall	Yes	Yes	Yes	Yes	Yes	Yes	Yes	Yes	Yes	Yes
Participant 3	Front Fall	Yes	Yes	Yes	Yes	Yes	Yes	Yes	Yes	Yes	Yes
	Back Fall	Yes	Yes	Yes	Yes	Yes	Yes	Yes	Yes	Yes	Yes
	Left Fall	Yes	Yes	Yes	Yes	Yes	Yes	**No**	**No**	Yes	Yes
	Right Fall	Yes	Yes	Yes	Yes	Yes	Yes	Yes	**No**	Yes	Yes
	Rotate Fall	Yes	Yes	Yes	Yes	Yes	Yes	Yes	Yes	Yes	Yes

## Data Availability

SmartallMM data can be found in https://anonymous.4open.science/r/smartfallmm-4588.
